# Revisiting the Construct Validity of the DASS-21 in Thai Young People: Evidence for a General Distress Factor from a Cross-Sectional Psychometric Study

**DOI:** 10.3390/nursrep16100361

**Published:** 2026-10-12

**Authors:** Nopporn Vongsirimas, Phunagphet Kesornsamut, Wareerat Thanoi, Sirada Kesornsri, Sudarat Pianchob, Sathaka Pimruen, Nirunya Narupan, Piyanee Klainin-Yobas

**Affiliations:** 1 Faculty of Nursing, Mahidol University, Bangkok 10700, Thailand; nopporn.von@mahidol.ac.th (N.V.); wareerat.tha@mahidol.ac.th (W.T.);; 2 Alice Lee Centre for Nursing Studies (NUS Nursing), Yong Loo Lin School of Medicine, National University of Singapore Level 2, Clinical Research Centre, 10 Medical Drive, Singapore 117597, Singapore; nurpk@nus.edu.sg

**Keywords:** DASS-21, Thai young people, factor analysis, construct validity, general psychological distress, mental health screening

## Abstract

**Background/Objectives:** The Depression, Anxiety, and Stress Scales–21 (DASS-21) is widely used to assess psychological distress, but its factor structure may vary across contexts. This study evaluated the factor structure and construct validity of the Thai DASS-21 among Thai young people. **Methods:** In this cross-sectional study, 220 Thai young people aged 12–24 years completed the DASS-21 and measures of psychological well-being, mindfulness, and suicide risk. Exploratory factor analysis used principal axis factoring with unrestricted, two-factor, and three-factor solutions; oblimin rotation was used for the specified solutions. FA-based parallel analysis was conducted. Confirmatory factor analysis of ordinal items compared one-factor, two-factor, and original three-factor models in the same sample. **Results:** EFA showed substantial common variance across items. Mean-eigenvalue parallel analysis suggested five factors, with a dominant first factor. Among the specified EFA solutions, the two-factor solution was more interpretable and explained 44.09% of the variance. CFA fit indices favored the two-factor Distress and Depression model over the one-factor model; fit was comparable to the original three-factor model, with fewer free parameters. Distress and Depression scores correlated negatively with psychological well-being (r = −0.54 and −0.71, respectively) and mindfulness (r = −0.73 and −0.64, respectively), and positively with suicide risk (r = 0.46 and 0.45, respectively; n = 71). **Conclusions:** The Thai DASS-21 showed a strong general distress component with partial differentiation into related Distress and Depression factors. Findings do not establish strict unidimensionality or justify replacing subscale scores with the total score. Independent replication is required.

## 1. Introduction

Adolescent mental health has emerged as an increasingly urgent global concern, with depression, anxiety, and stress ranking among the most prevalent psychological conditions affecting this age group [1]. These emotional challenges have profound implications for academic functioning, psychosocial development, and long-term well-being [2]. In Southeast Asia, recent studies have shown rising psychological distress among adolescents, especially in post-pandemic contexts marked by educational disruptions and social instability During the COVID-19 pandemic, school closures as part of broader social lockdown measures were associated with adverse mental health symptoms, including psychological distress and anxiety, among children and adolescents [3,4].

In Thailand, a previous validation study evaluated the DASS-21 among nursing students in an online-learning context [5]. This evidence does not establish whether the original three-factor structure is reproduced among Thai young people aged 12–24 years who are enrolled across secondary-school and university settings. The DASS-21, the short-form version of the original 42-item scale [6], has gained international recognition because of its conceptual clarity and psychometric performance across settings [7]. It comprises three theoretically distinct but related subscales: depression, anxiety, and stress. Although studies have supported its factorial validity in diverse populations, including adults, evidence in adolescent and young-adult samples remains comparatively limited, particularly in collectivist and non-Western contexts where the expression of psychological distress may differ from that in the setting where the scale was developed [8,9].

Cultural and developmental factors may shape how young people perceive, interpret, and express emotional distress. In Thailand, social norms that emphasize emotional restraint, respect for others, and social harmony may influence the disclosure of internalizing symptoms, particularly in school settings where conformity and group cohesion are reinforced [10,11]. These contextual considerations underscore the need for rigorous construct validation before the DASS-21 is applied to young people outside Western contexts.

To address these gaps, the present study examined the construct validity of the Thai version of the DASS-21 among Thai young people aged 12–24 years. The research question was whether the latent structure of the DASS-21 in this sample was more consistent with a general distress factor or with the originally proposed three-factor model of depression, anxiety, and stress. Exploratory factor analysis (EFA) was used to examine the underlying item structure, and confirmatory factor analysis (CFA) was used to compare the competing models within the same sample. Clarifying the dimensional structure of the DASS-21 in this population may inform score interpretation and strengthen the evidence base for mental health research among young people.

## 2. Materials and Methods

### 2.1. Study Design and Participants

This cross-sectional methodological study was conducted as part of a broader research initiative entitled “The Development Model to Promote Psychological Well-being and Prevent Mental Health Problems in Children and Adolescents.”

The target population was Thai young people aged 12–24 years, a range that spans adolescence and young adulthood. The lower age boundary of 12 years corresponds to entry into the first year of lower secondary school in Thailand. Participants were recruited from public secondary schools and a public university located in Bangkok and the surrounding metropolitan area using a multistage cluster sampling procedure.

For the secondary-school component, eligible institutions were public schools offering education from Mathayom 1 to Mathayom 6 and having sufficient student enrollment to support classroom-based cluster sampling. Within each participating school, the sampling frame comprised classrooms from Mathayom 1 to Mathayom 6, with approximately 10 classrooms per grade and approximately 35 students per classroom. Classrooms were selected by drawing lots, and all eligible students in the selected classrooms were invited to participate.

For the university component, one public university was randomly selected from eligible public universities in Bangkok and the surrounding metropolitan area. Within the selected university, three faculties were randomly selected from a total of 36 faculties. Eligible students from the selected faculties were then invited to participate according to the study inclusion criteria. Institutional permission was obtained before participant recruitment.

Eligibility criteria were: (1) Thai nationality, (2) current enrollment in an educational institution, and (3) written informed consent from participants and, where applicable, their parents or legal guardians. Individuals with a self-reported history of clinically diagnosed psychiatric disorders were excluded because the main aim was to characterize the measurement in a nonclinical community sample [1]. Furthermore, we aimed to avoid confounding effects associated with psychiatric disorders, such as stress, treatment effects, and medication effects. Findings from this study cannot be generalized to people with psychiatric disorders.

Data were collected between 2022 and 2023. A total of 220 eligible students completed the questionnaire and were included in the analyses. The number of students invited to participate, the number who declined participation, and the number who did not return the questionnaire were not recorded. Therefore, a response rate could not be calculated, and the extent or characteristics of non-participation could not be assessed.

The sample size was determined using established recommendations for factor analysis, which suggest a minimum of 5–10 participants per item and an overall sample size of at least 200 to support the stability of an exploratory factor solution [12,13,14]. Given that the DASS-21 consists of 21 items, the sample of 220 participants was considered sufficient for the EFA. The CFA was conducted as a within-sample comparison of competing models and did not provide an independent confirmation of the EFA results [2].

### 2.2. Instrument

The Thai-translated version of the Depression Anxiety Stress Scales (DASS-21) was used, with permission from the original authors [6]. The Thai version of the DASS-21 was translated by Sukanlaya Sawang and colleagues (2013) using a back-translation procedure and subsequently adapted for use in Thai populations by Nuttorn Pityaratstian and Napakkawat Buathong [15]. The adaptation involved modifying the wording to ensure its appropriateness and comprehensibility within the Thai cultural and community context. The Thai version has demonstrated acceptable internal consistency, with Cronbach’s alpha coefficients of 0.82 for Depression, 0.78 for Anxiety, and 0.69 for Stress. The scale contains 21 items rated on a 4-point Likert scale (0 = did not apply to me at all to 3 = applied to me very much or most of the time). Scores for each subscale (depression, anxiety, and stress) were multiplied by two to align with the full 42-item scale.

In addition to the DASS-21, psychological well-being, mindfulness, and suicide risk were assessed as external constructs.

Psychological well-being was assessed using the 18-item Psychological Well-Being Scale (PWBS) [16]. Items are rated on a 6-point response scale from 1 (strongly disagree) to 6 (strongly agree), yielding total scores from 18 to 108. Higher scores indicate better psychological well-being. The Thai version was developed through a back-translation procedure and has undergone psychometric evaluation in Thai samples [17]. In a previous Thai adolescent sample, the PWBS demonstrated acceptable internal consistency (Cronbach’s alpha = 0.77) [18].

Mindfulness was assessed using the 15-item Mindful Attention Awareness Scale (MAAS) [19]. Each item is rated on a 6-point response scale from 1 (almost always) to 6 (almost never), with total scores ranging from 15 to 90. Higher scores indicate greater dispositional mindfulness. The MAAS has been examined in Thai samples [20] and demonstrated acceptable internal consistency in a previous Thai adolescent sample (Cronbach’s alpha = 0.84) [18].

Suicide risk was assessed using the 8-question suicide-risk assessment (8Q) developed by the Department of Mental Health, Ministry of Public Health, Thailand [21]. The instrument assesses death wishes, self-harm, suicidal thoughts, inability to control suicidal thoughts, suicide plans, preparation for suicide, self-injury, suicide attempts during the past month, and lifetime history of suicide attempts. Items are scored using weighted yes/no responses, with higher total scores indicating greater suicide risk. According to the Department of Mental Health guidance, scores of 1–8, 9–16, and 17 or higher indicate low, moderate, and severe suicide risk, respectively.

### 2.3. Procedure

The study protocol was approved by the institutional ethics committee (IRB-NS2019/526.0912). Trained researchers administered paper-based questionnaires in classroom settings. Participants completed the DASS-21 anonymously within 10–15 min. Participation was voluntary, and questionnaire responses were handled confidentially. Researchers answered participants’ questions as needed.

### 2.4. Data Analysis

Analyses were conducted using JASP Statistics version 0.98.1. Exploratory factor analysis (EFA) used principal axis factoring to examine the common latent structure of the 21 DASS items. Sampling adequacy was assessed using the Kaiser–Meyer–Olkin measure and Bartlett’s test of sphericity. The EFA was undertaken in three stages. First, an unrestricted solution was examined without pre-specifying the number of factors. Second, a two-factor solution was specified. Third, a three-factor solution was specified to evaluate the original depression, anxiety, and stress framework. Oblimin rotation was applied to the specified two-factor and three-factor solutions because the factors were expected to correlate. Pattern coefficients with an absolute magnitude of 0.40 or greater were considered salient, and weak loadings, cross-loadings, and uniqueness values were examined when interpreting each solution.

Factor-analysis-based parallel analysis (PA) was performed as an additional factor-retention criterion. Observed eigenvalues were compared with the mean eigenvalues obtained from simulated datasets with the same sample size and number of items. A factor was retained when its observed eigenvalue exceeded the corresponding mean simulated eigenvalue. The PA results were considered alongside the unrestricted EFA, scree plot, theoretical framework, and interpretability of the two- and three-factor solutions. Because PA based on mean simulated eigenvalues did not specifically support either a two- or three-factor solution, it was not used alone to select the final factor structure.

Additionally, confirmatory factor analysis (CFA) was conducted in the same sample to compare the factorial structure of the DASS-21. This analysis was performed in JASP Statistics Software (JASP team, 2026). Given that the measurement items were ordinal, the estimation used weighted least squares with mean- and variance-adjusted test statistics (WLSMV). The CFA was interpreted as a within-sample comparison of the competing models rather than as independent confirmation of the EFA. Model fit was evaluated using chi-square statistics per degree of freedom, the comparative fit index (CFI), Tucker–Lewis index (TLI), root mean square error of approximation (RMSEA), and standardized root mean square residual (SRMR) [13,22]. Values of 0.90 or above for CFI and TLI were interpreted as acceptable fit. For RMSEA and SRMR, values below 0.08 indicated acceptable fit, whereas values below 0.06 suggested good fit [13,23]. Standardized factor loadings were examined to assess item-factor relationships, with loadings of 0.40 or higher considered meaningful. Modification indices and standardized residuals were reviewed to identify localized strain, although model modifications were made only when theoretically justifiable [13,22].

### 2.5. Ethical Considerations

The study protocol was approved by the Research Ethics Committee of Mahidol University (IRB-NS2019/526.0912) and conducted in accordance with the Declaration of Helsinki. Written informed consent was obtained from each participant and, for participants who were minors, from a parent or legal guardian before data collection. Participation was voluntary, and responses were anonymous and handled confidentially.

## 3. Results

### 3.1. Exploratory Factor Analysis

A total of 220 young people participated in the study, with a mean age of 17.32 years (SD = 2.23). The sample was predominantly female (68.6%). As the sample included participants aged 12–24 years, it encompassed both adolescence and young adulthood. The suitability of the data for factor analysis was confirmed. The Kaiser–Meyer–Olkin (KMO) measure was 0.911, indicating excellent sampling adequacy, and Bartlett’s test of sphericity was statistically significant (χ^2^ = 2258.569, df = 210, *p* < 0.001), supporting the factorability of the correlation matrix.

The EFA was conducted in three stages. First, the factor structure was explored without pre-specifying the number of factors. Second, a two-factor solution was specified. Third, a three-factor solution was specified. Oblique (oblimin) rotation was used for the specified solutions because the DASS dimensions were expected to correlate. FA-based parallel analysis was used as an additional retention criterion.

#### 3.1.1. Unrestricted Analysis and Parallel Analysis

The unrestricted analysis was used to examine the number of factors suggested by the data. Parallel analysis compared observed eigenvalues with mean eigenvalues from simulated data of the same dimensions. The observed eigenvalues exceeded the simulated mean eigenvalues for Factors 1 through 5. The first factor was markedly larger than the remaining factors (8.009 versus 1.081 for the second factor), indicating a strong general distress dimension. Parallel analysis therefore did not identify exactly two or three factors for retention. The results are presented in Table 1.

#### 3.1.2. Specified Two-Factor Solution

A two-factor solution was then specified to evaluate a parsimonious alternative. The exact-fit test was significant, chi-square(169) = 409.925, *p* < 0.001. The first two unrotated eigenvalues were 8.071 and 1.188, and the solution explained 44.09% of the total variance. After rotation, Factor 1 and Factor 2 accounted for 23.06% and 21.03% of the variance, respectively.

The pattern matrix supported a mixed stress/anxiety factor and a depressive symptom factor. Factor 1 was defined by panic, fear, agitation, over-reactivity, nervous energy, touchiness, trembling, and heart-action items. Factor 2 was defined by down-heartedness, anhedonia, meaninglessness, low initiative, low self-worth, lack of enthusiasm, and lack of future orientation. The factors were moderately correlated (r = 0.638). Q2 and Q4 did not reach a 0.40 loading on either factor, and Q12 had a borderline loading (0.395) on Factor 1. The results are presented in Table 2.

#### 3.1.3. Specified Three-Factor Solution

A three-factor solution was specified to evaluate the original depression, anxiety, and stress framework. The solution yielded a mixed stress/anxiety factor (Factor 1), a depressive symptom factor (Factor 2), and a small somatic-anxiety factor (Factor 3). Factor 1 contained eight salient items, including panic, fear, agitation, over-reactivity, nervous energy, and touchiness. Factor 2 contained seven salient depressive items. Factor 3 was defined only by Q19 and Q4. Q1, Q7, and Q12 did not show a salient loading, and Q2 loaded weakly on Factor 2. The three-factor solution therefore did not reproduce the intended DASS-21 structure. The results are presented in Table 3.

#### 3.1.4. Comparison of Specified Solutions

Overall, the EFA results showed a dominant general distress dimension and did not reproduce the original three-factor DASS-21 structure. The two-factor solution was more interpretable than the specified three-factor solution, although it should not be described as selected by the present parallel analysis (Table 4). The final decision regarding the two-factor structure should be based on the planned comparison of competing CFA models. 

### 3.2. Confirmatory Factor Analysis (CFA)

To further evaluate the construct validity of the DASS-21, confirmatory factor analysis (CFA) was performed using JASP Statistics Software (JASP team, 2026). The WLSMV model was an estimator given that the data were ordinal. Based on EFA results, three competing models were tested: (1) a unidimensional model representing the general distress factor, (2) a two-factor structure representing the distress and depression factor, and (3) the original three-factor model consisting of Depression, Anxiety, and Stress.

The one-factor model shows the worst fit with the sample data. Most fit indices achieved minimally acceptable thresholds (χ^2^/df = 2.73, CFI = 0.93, TLI = 0.92, and GFI = 0.98). However, the RMSEA (0.09) exceeded the acceptable ceiling of 0.08, and its confidence interval (0.08–0.09) was above the acceptable boundary.

The two-factor model shows the best-overall fit with the lowest χ^2^/df (1.92), the strongest fit indices (CFI = 0.96, TLI = 0.96, RMSEA= 0.06, 90% CI of RMSEA= 0.05–0.07), and good residual-based fit (SRMR = 0.07). All indices were within the “good fit” range. The two-factor model shows the most parsimonious structure of the models.

The three-factor model also showed good fits for most indices (CFI = 0.96, TLI = 0.96, GFI = 0.99). Nonetheless, χ^2^/df (1.94) and RMSEA (0.07, CI = 0.06–0.08) were slightly worse than the two-factor model. Notably, the three-factor model had fewer degrees of freedom (df) but slightly higher χ^2^ (361.00) higher than the two-factor model (χ^2^ (360.40)). This evidence suggests that adding more parameters did not improve model fits.

Chi-square difference tests (Δχ^2^) were performed to compare fits across the three nested models. Specifically, the comparison between the one-factor and two-factor model confirmed that the two-factor model had significantly better fits that the one-factor model (Δχ^2^(1) = 155.15, *p* < 0.001). Moreover, results suggested that the three-factor model did not yield significant improvement in fits, in comparison with the two-factor model (Δχ^2^(2) = 0.60, *p* > 0.05). Given its most parsimonious structure and acceptable fit indices, the two-factor model was retained and considered the best solution for the Thai-version DASS 21. Additionally, the JASP output provided a heterotrait–monotrait ratio of 0.78, against the threshold value of 0.90. This evidence suggested that the two factors clearly capture different constructs, supporting discriminant validity of the DASS-21. The results are presented in Table 5.

Given the items loaded in the two factors, we labeled the first factor as “Distress” and the second factor as “Depression.” All factor loadings achieved statistical significance (*p* < 0.001) (Table 6). The Distress factor had factor loadings ranging from 0.56 to 0.87 and the Depression factor had factor loadings ranging from 0.67 to 0.90. Cronbach’s alpha was 0.88 and 0.89, for the Distress and Depression factor respectively, suggesting good internal consistency reliability. Additionally, the distress factor had a significant correlation (r = 0.69, *p* < 0.001) with the depression factor. The results are presented in Table 6.

#### Convergent and Discriminant Analysis

Convergent and Discriminant validity of the Thai-version DASS were tested using its correlation with theoretically related variables (Table 7). As anticipated, the distress factor had a significant negative correlation with psychological well-being (−0.54, *p* < 0.001) and mindfulness (−0.73, *p* < 0.001), and had a significant positive association with suicide risk (0.46, *p* < 0.001). Similarly, the depression factor had a significant negative correlation with psychological well-being (−0.71, *p* < 0.001) and mindfulness (−0.64, *p* < 0.001), and had a significant positive association with suicide risk (0.45, *p* < 0.001). These findings further support the construct validity of the DASS-21. The results are presented in Table 7.

## 4. Discussion

The present study evaluated the construct validity of the DASS-21 among Thai young people using complementary exploratory and confirmatory analyses. The EFA did not reproduce the original depression, anxiety, and stress structure. The CFA showed that the two-factor model fit the data better than the one-factor model and was slightly more parsimonious than the original three-factor model. Taken together, the findings are most consistent with a strong general distress component and partial differentiation into two related dimensions, rather than strict unidimensionality or three clearly distinct symptom domains. The unrestricted EFA and parallel analysis provide important context for this conclusion. In the FA-based parallel analysis using mean simulated eigenvalues, the first five observed eigenvalues exceeded their simulated counterparts, and the first eigenvalue was much larger than the second. This pattern indicates substantial common variance across the DASS-21 items, but it does not select either a two-factor or a three-factor solution. The specified two-factor and three-factor EFAs were therefore evaluated as theoretically informed comparative models, rather than as structures selected by parallel analysis alone.

Among the specified EFA solutions, the two-factor pattern was more interpretable. Stress and anxiety items formed a combined distress factor, whereas most depressive items formed a second factor. The two factors were moderately correlated (r = 0.64). In the specified three-factor solution, the proposed anxiety and stress items did not separate as intended, and the third factor was defined by only two somatic-anxiety items. Several items also lacked a salient loading or loaded weakly. These findings limit support for the original three-subscale configuration in this sample.

The CFA provided the basis for selecting the two-factor model for this study. Its fit was acceptable to good—chi-square (188) = 360.402, CFI = 0.96, TLI = 0.96, RMSEA = 0.06, and SRMR = 0.07—whereas the one-factor model fit less well. The original three-factor CFA had a very similar overall fit, but its anxiety and stress factors were extremely highly correlated (r = 0.98), which indicates limited empirical separation of these two dimensions. In the two-factor CFA, the Distress and Depression factors were strongly related (r = 0.69) but remained distinguishable. The allocation of the dryness-of-mouth item to the Depression factor should be interpreted cautiously because it did not show a salient loading in the two-factor EFA.

Psychometric studies have reported that the DASS-21 factor structure varies across populations and developmental groups [24,25]. The present findings may reflect differences in symptom expression, item interpretation, language, or sample characteristics. However, this study did not measure cultural values, emotional expression, or self-construal. Cultural explanations should therefore be considered hypotheses for future research rather than explanations established by the present data.

The reported inverse associations with psychological well-being and mindfulness, together with the positive association with suicide risk, are consistent with the expected external correlates of emotional distress. These associations provide preliminary nomological support for the measure. They do not establish discriminant validity between the Distress and Depression factors, and they do not determine whether a total score or subscale scores should be preferred.

For nursing and school-health applications, the DASS-21 should continue to be treated as a self-report symptom-screening instrument rather than a diagnostic measure [6]. In this sample, the total score can be used as an indicator of broad psychological distress. If subscale scores are reported, their theoretical basis and the observed overlap between stress and anxiety should be acknowledged. The results do not support using a DASS-21 score alone to label a mental disorder or to make decisions about diagnosis.

Several limitations require consideration. The cross-sectional sample was drawn from central Thailand and included young people aged 12–24 years, which may limit generalizability and introduce developmental heterogeneity. The predominance of female participants (68.6%) may also limit the generalizability of the findings to male students and students of other gender identities. In addition, the exclusion of participants with self-reported clinically diagnosed psychiatric disorders may limit the applicability of the findings to young people with psychiatric disorders. In addition, because non-participation could not be assessed, the potential influence of selection bias on the findings cannot be determined. EFA and CFA were conducted in the same sample, so the CFA represents a within-sample comparison rather than independent confirmation. Future studies should cross-validate the two-factor solution in independent samples, apply a 95th-percentile parallel-analysis criterion, examine bifactor or exploratory structural equation models, and test measurement invariance across age groups, gender, and regions.

## 5. Conclusions

The psychometric evaluation of the DASS-21 among Thai young people provides preliminary evidence of a dominant general psychological distress factor. Although exploratory and confirmatory analyses indicated substantial common variance and high inter-factor correlations, the three-factor model also showed acceptable fit. These findings do not establish strict unidimensionality or the superiority of the total score over the depression, anxiety, and stress subscale scores.

The present study did not directly measure cultural values, emotional expression, or self-construal. Cultural explanations for the observed factor pattern should therefore be treated as hypotheses for future research rather than as conclusions from the current analyses.

The DASS-21 is a self-report screening and symptom-assessment instrument, not a diagnostic tool [6]. This study did not test diagnostic accuracy, clinical cut-offs, or misclassification. The total score may be reported as a broad distress indicator in similar research samples, but further independent evidence is needed before recommending that it replace the depression, anxiety, and stress subscale scores.

## Figures and Tables

**Table 1:** FA-based parallel analysis from the unrestricted exploratory analysis.

Factor	Observed Eigenvalue	Mean Simulated Eigenvalue	Retention Result
1	8.009	0.754	Retain
2	1.081	0.526	Retain
3	0.462	0.412	Retain
4	0.398	0.352	Retain
5	0.336	0.294	Retain
6	0.229	0.244	Do not retain

Note. Factors 7 through 20 did not meet the mean-eigenvalue criterion.

**Table 2:** Pattern matrix and uniqueness for the specified two-factor solution.

Item	Item Content	Factor 1	Factor 2	Uniqueness
Q15	I felt I was close to panic (anxiety)	0.820	−0.159	0.469
Q20	I felt scared without any good reason (anxiety)	0.678	0.061	0.484
Q9	I was worried about situations in which I might panic and make a fool of myself (anxiety)	0.641	0.107	0.489
Q11	I found myself getting agitated (stress)	0.624	0.216	0.392
Q6	I tended to over-react to situations (stress)	0.605	−0.031	0.657
Q14	I was intolerant of anything that kept me from getting on with what I was doing (stress)	0.604	−0.069	0.683
Q8	I felt that I was using a lot of nervous energy (stress)	0.586	0.173	0.498
Q18	I felt that I was rather touchy (stress)	0.573	−0.039	0.698
Q7	I experienced trembling (anxiety)	0.462	0.134	0.690
Q19	I was aware of the action of my heart in the absence of physical exertion (anxiety)	0.461	0.041	0.761
Q1	I found it hard to wind down (stress)	0.415	0.273	0.609
Q12	I found it difficult to relax (stress)	0.395	0.218	0.686
Q4	I experienced breathing difficulty (anxiety)	0.277	0.341	0.687
Q2	I was aware of dryness of my mouth (anxiety)	0.259	0.382	0.661
Q13	I felt down-hearted and blue (depress)	0.188	0.598	0.463
Q3	I could not experience any positive feeling at all (depress)	0.163	0.657	0.405
Q21	I felt that life was meaningless (depress)	−0.111	0.911	0.287
Q5	I found it difficult to work up the initiative to do things (depress)	0.081	0.568	0.612
Q17	I felt I was not worth much as a person (depress)	0.054	0.766	0.357
Q16	I was unable to become enthusiastic about anything (depress)	0.050	0.610	0.587
Q10	I felt that I had nothing to look forward to (depress)	−0.038	0.684	0.564

Note. Factor 1 was interpreted as a mixed stress/anxiety dimension and Factor 2 as a depressive symptom dimension. Pattern coefficients are shown. Oblimin rotation was applied. Values of 0.40 or greater were treated as salient for interpretation.

**Table 3:** Pattern matrix for the specified three-factor solution.

Item	Item Content	Factor 1	Factor 2	Factor 3
Q15	I felt I was close to panic (anxiety)	0.837	0.108	−0.048
Q9	I was worried about situations in which I might panic and make a fool of myself (anxiety)	0.680	−0.160	−0.085
Q6	I tended to over-react to situations (stress)	0.618	−0.016	−0.048
Q8	I felt that I was using a lot of nervous energy (stress)	0.574	−0.212	−0.006
Q14	I was intolerant of anything that kept me from getting on with what I was doing (stress)	0.568	0.039	0.039
Q20	I felt scared without any good reason (anxiety)	0.561	−0.073	0.178
Q11	I found myself getting agitated (stress)	0.526	−0.231	0.144
Q18	I felt that I was rather touchy (stress)	0.489	0.029	0.130
Q12	I found it difficult to relax (stress)	0.347	−0.232	0.067
Q7	I experienced trembling (anxiety)	0.300	−0.109	0.281
Q1	I found it hard to wind down (stress)	0.285	−0.262	0.215
Q21	I felt that life was meaningless (depress)	−0.133	−0.895	0.050
Q17	I felt I was not worth much as a person (depress)	−0.008	−0.749	0.115
Q10	I felt that I had nothing to look forward to (depress)	0.000	−0.702	−0.072
Q3	I could not experience any positive feeling at all (depress)	0.039	−0.628	0.222
Q16	I was unable to become enthusiastic about anything (depress)	0.059	−0.623	−0.024
Q5	I found it difficult to work up the initiative to do things (depress)	0.135	−0.608	−0.119
Q13	I felt down-hearted and blue (depress)	0.152	−0.601	0.058
Q2	I was aware of dryness of my mouth (anxiety)	0.175	−0.373	0.142
Q19	I was aware of the action of my heart in the absence of physical exertion (anxiety)	0.125	0.104	0.705
Q4	I experienced breathing difficulty (anxiety)	−0.051	−0.258	0.594

Note. Pattern coefficients are retained as displayed in the supplied output. The sign of a factor is arbitrary; the negative coefficients on Factor 2 identify the depressive symptom pole. The three-factor output used principal axis factoring with oblimin rotation. Values with an absolute magnitude of 0.40 or greater were treated as salient.

**Table 4:** Summary of the exploratory factor analyses.

Analysis	Main Finding	Interpretation
Unrestricted EFA with parallel analysis	Factors 1–5 exceeded mean simulated eigenvalues; Factor 1 was dominant.	Parallel analysis did not select exactly two or three factors.
Specified two-factor EFA	Mixed stress/anxiety and depressive symptom factors; r = 0.638; 44.09% total variance.	Most interpretable parsimonious alternative among the specified solutions.
Specified three-factor EFA	Mixed stress/anxiety, depressive symptoms, and a two-item somatic-anxiety factor.	Did not recover the original depression, anxiety, and stress structure.

**Table 5:** Goodness-of-Fit Indices for Competing Models of the DASS-21 (n = 220).

Model	χ^2^	df	*p*	χ^2^/df	CFI	TLI	RMSEA	90% RMSEA	SRMR	GFI
One-factor Model	515.55	189	<0.001	2.73	0.93	0.92	0.09	0.08–0.09	0.08	0.98
Two-factor Model	360.40	188	<0.001	1.92	0.96	0.96	0.06	0.05–0.07	0.07	0.99
Three-factor Model	361.00	186	<0.001	1.94	0.96	0.96	0.07	0.06–0.08	0.07	0.99

Note: This CFA used WLSMV as an estimator. χ^2^/df = Chi-square per degree of freedom, CFI = Comparative Fit Index, TLI = Tucker–Lewis Index, RMSEA = Root Mean Square Error of Approximation, 90% RMSEA = 90% confidence interval around RMSEA, SRMR = Standardized Root Mean Square Residual, GFI = Goodness of Fit index.

**Table 6:** CFA results of the two-factor model.

Observed Variables	Factor Loading (λ)	
	Factor 1 Distress	Factor 2 Depression
Q4: Breathing difficulty (Anxiety)	0.67 ***	
Q12: Difficult to relax (Stress)	0.66 ***	
Q1: Hard to wind down (Stress)	0.72 ***	
Q19: Heart action (Anxiety)	0.56 ***	
Q7: Trembling (Anxiety)	0.63 ***	
Q18: Touchy (Stress)	0.57 ***	
Q8: Nervous energy (Stress)	0.77 ***	
Q14: Intolerant (Stress)	0.59 ***	
Q6: Over-react (Stress)	0.63 ***	
Q11: Agitated (Stress)	0.87 ***	
Q9: Panic/Fool of myself (Anxiety)	0.77 ***	
Q20: Scared for no reason (Anxiety)	0.79 ***	
Q15: Close to panic (Anxiety)	0.72 ***	
Q3: No positive feeling (Depression)		0.87 ***
Q17: Not worth much (Depression)		0.88 ***
Q13: Down-hearted and blue (Depression)		0.83 ***
Q21: Life meaningless (Depression)		0.90 ***
Q16: Not enthusiastic (Depression)		0.68 ***
Q2: Dryness of mouth (Anxiety)		0.67 ***
Q5: Difficult to work up initiative (Depression)		0.67 ***
Q10: Nothing to look forward to (Depression)		0.72 ***
Coefficient Alpha	0.88	0.89

Note: *** Significant at the level of 0.001 (2-tailed).

**Table 7:** Discriminant and Convergent Relationships among variables.

	Psychological Well-Being (*n* = 220)	Mindfulness (*n* = 220)	Suicide Risk (*n* = 71)
The Distress Factor	−0.54 ***	−0.73 ***	0.46 ***
The Depression Factor	−0.71 ***	−0.64 ***	0.45 ***

Note: *** Significant at the level of 0.001 (2-tailed).

## Data Availability

The datasets generated and/or analyzed during the current study are not publicly available due to the sensitive nature of young people’s mental health data and the ethical constraints of the institutional review board. The data are available from the corresponding author on reasonable request and with permission from the Faculty of Nursing, Mahidol University.

## Public Involvement Statement

There was no public or patient involvement in the design, conduct, reporting, or dissemination planning of this study. Young people were involved solely as research participants.

## Guidelines and Standards Statement

This manuscript was prepared in accordance with the Strengthening the Reporting of Observational Studies in Epidemiology (STROBE) guidelines for cross-sectional studies [26].

## Use of Artificial Intelligence

During the preparation of this manuscript, the authors used ChatGPT (OpenAI; https://chatgpt.com/; accessed on 1 October 2026) solely to assist with English language editing, including grammar, wording, clarity, and consistency, in text written by the authors. ChatGPT was not used for data analysis, interpretation of findings, or generation of scientific conclusions. The authors critically reviewed and verified all suggested edits and take full responsibility for the accuracy and integrity of the final manuscript.

## Abbreviations

AGFI: Adjusted Goodness of Fit Index; CFA: Confirmatory Factor Analysis; CFI: Comparative Fit Index; DASS-21: Depression Anxiety Stress Scales–21 items; EFA: Exploratory Factor Analysis; GFI: Goodness of Fit Index; IRB: Institutional Review Board; KMO: Kaiser–Meyer–Olkin measure of sampling adequacy; NRCT: National Research Council of Thailand; PAF: Principal Axis Factoring; RMSEA: Root Mean Square Error of Approximation; SRMR: Standardized Root Mean Square Residual; TLI: Tucker–Lewis Index.
